# *Streptococcus thermophilus* Biofilm Formation: A Remnant Trait of Ancestral Commensal Life?

**DOI:** 10.1371/journal.pone.0128099

**Published:** 2015-06-02

**Authors:** Benoit Couvigny, Claire Thérial, Céline Gautier, Pierre Renault, Romain Briandet, Eric Guédon

**Affiliations:** 1 INRA, UMR 1319 Micalis, Domaine de Vilvert, F-78352 Jouy-en-Josas, France; 2 AgroParisTech, UMR MICALIS, Jouy-en-Josas, France; University Hospital of the Albert-Ludwigs-University Freiburg, GERMANY

## Abstract

Microorganisms have a long history of use in food production and preservation. Their adaptation to food environments has profoundly modified their features, mainly through genomic flux. *Streptococcus thermophilus*, one of the most frequent starter culture organisms consumed daily by humans emerged recently from a commensal ancestor. As such, it is a useful model for genomic studies of bacterial domestication processes. Many streptococcal species form biofilms, a key feature of the major lifestyle of these bacteria in nature. However, few descriptions of *S*. *thermophilus* biofilms have been reported. An analysis of the ability of a representative collection of natural isolates to form biofilms revealed that *S*. *thermophilus* was a poor biofilm producer and that this characteristic was associated with an inability to attach firmly to surfaces. The identification of three biofilm-associated genes in the strain producing the most biofilms shed light on the reasons for the rarity of this trait in this species. These genes encode proteins involved in crucial stages of biofilm formation and are heterogeneously distributed between strains. One of the biofilm genes appears to have been acquired by horizontal transfer. The other two are located in loci presenting features of reductive evolution, and are absent from most of the strains analyzed. Their orthologs in commensal bacteria are involved in adhesion to host cells, suggesting that they are remnants of ancestral functions. The biofilm phenotype appears to be a commensal trait that has been lost during the genetic domestication of *S*. *thermophilus*, consistent with its adaptation to the milk environment and the selection of starter strains for dairy fermentations.

## Introduction

Adaptation to their external environment is crucial for the survival and replication of bacteria. The modulation of gene expression is one of the major adaptive strategies by which bacteria deal with changing environmental conditions. However, long-term adaptation, during the colonization of a new habitat for example, requires more profound genomic modifications. These changes include both the acquisition of new gene sets and the discarding of genes that are no longer useful, by gene loss and/or gene inactivation [1, 2]. Niche adaptation is a major driving force shaping the bacterial genome [3–5]. The availability of complete genome sequences for closely related species, such as the many streptococcal genomes currently available, provides a remarkable opportunity for detecting niche-specific adaptation by comparative evolutionary genomics [6–12]. The genus *Streptococcus* contains diverse species, most of which are commensal or pathogenic in humans and animals [13]. They occupy a broad range of ecological niches within the host, and the factors governing niche colonization remain poorly understood. The importance of these bacteria as etiological agents of numerous infections has led to studies investigating their adaptive versatility, at the genome level in particular.

*Streptococcus thermophilus* is the only species of this genus to be widely used as a starter in the dairy industry and to have the “Generally Regarded As Safe” status. It belongs to the *salivarius* group of the Viridians streptococci [13], which includes two other species, *Streptococcus salivarius* and *Streptococcus vestibularis*. These two species are both commensal bacteria of the human gut, whereas the environmental reservoir of *S*. *thermophilus* has not been identified [14]. It grows spontaneously in traditional dairy products and is believed to persist in the farm environment [15–17]. Multilocus sequence typing and comparative genomic analysis have revealed that there is little polymorphism in the *S*. *thermophilus* population, and that this species displays significant allelic divergence from the other two species of the *salivarius* group [14, 18, 19]. *S*. *thermophilus* is a clonal species that emerged only recently on the evolutionary timescale (3,000–30,000 years ago), from a commensal ancestor of the *salivarius* group [20]. Its adaptation to a narrow and well defined niche (milk) has shaped its genome through loss-of-function events and horizontal gene transfer (HGT) [21–23]. Approximately 10% of the ORFs of *S*. *thermophilus* are pseudogenes, their original functions being unnecessary for growth in milk. Many of these pseudogenes encoded proteins involved in carbohydrate metabolism, a function not particularly useful in milk, which contains few carbon sources [22, 23]. Commensal and pathogenic streptococci display numerous proteins at their surface, many of which have virulence-related functions. *S*. *thermophilus* has lost almost all of these features [21, 23], suggesting that direct contact with the host may be required for the maintenance of such functions. HGT events have contributed substantially to the genomic plasticity, population evolution and adaptation of this species to the milk environment. The genomic regions acquired include those encoding industrially important phenotypic traits, such as the production of bacteriocin, lantibiotics and exopolysaccharides, restriction-modification systems, oxygen tolerance, amino-acid metabolism and milk-protein degradation [19, 21–26].

Bacteria rarely live as planktonic organisms in their natural habitats. Instead, they are mostly organized into biofilms, composed of surface-associated multicellular communities encased in a self-produced matrix. Biofilms are ubiquitous in natural environments and are an inevitable component of hospital and industrial settings [27–29]. Many bacteria adopt a biofilm lifestyle, to enable them to survive and persist in hostile environments [27, 28, 30, 31], and biofilm formation is an important trait in many streptococcal species. Nevertheless, little is currently known about the ability of *S*. *thermophilus* to form biofilms and to live in them [32, 33]. We therefore evaluated the ability of a representative collection of natural isolates of *S*. *thermophilus* to form biofilms on an abiotic surface. We also used a genome-wide mutagenesis approach to identify genes associated with biofilm formation in the strain producing the most biofilm. Three biofilm-associated genes encoding proteins involved in the early stages of biofilm formation (adhesion to the substrate and matrix production) were identified. We characterized their functions and analyzed their distribution in the *S*. *thermophilus* isolates analyzed. Most *S*. *thermophilus* strains are poor biofilm producers, mostly because they have lost these traits, consistent with their adaptation to the milk environment and selection as starters for dairy fermentations.

## Materials and Methods

### Bacterial strains, plasmids, growth conditions, and DNA manipulation

The bacterial strains used in this study are listed in Table 1 and S1 Table. *Escherichia coli* TG1repA was used for plasmid propagation. *E*. *coli* was grown in Luria-Bertani medium at 37°C [34]. *S*. *thermophilus* strains were grown at 42°C in M17 medium containing 1% lactose, without shaking. *S*. *salivarius* strains were grown at 37°C in M17 medium containing 1% glucose, without shaking. When required, 5-bromo-4-chloro-3-indolyl-β-D-galactoside (0.04 g/l), isopropyl 1-thio-β-D-galactopyranoside (IPTG; 0.04 g/l), ampicillin (100 μg/ml for *E*. *coli*), erythromycin (4 μg/ml for *S*. *thermophilus* and *S*. *salivarius*, 100 μg/ml for *E*. *coli*) were added to the culture medium. Solid agar plates were prepared by adding 2% (wt/vol) agar to the medium. Standard molecular biology techniques were used and *E*. *coli* was electrotransformed as previously described [35]. Electrocompetent *S*. *thermophilus* cells were prepared as previously described [36]. PCR was performed with Fhusion high-fidelity DNA polymerase (NEB, MA, USA), in a GeneAmp 2400 PCR system (Applied Biosystems, Foster City, CA). The primers were purchased from Eurofins MWG Operon (Germany) and are listed in S1 Table.

**Table 1:** Characteristics of the *Streptococcus thermophilus* natural isolates used in this study.

Strain	ST^a^	Location	Source	Year	Biofilm status^b^	0171^c^	0714^c^	1361^c^
JIM8232	2	France	Milk	2002	+	+	+	+
CNRZ1575	16	Italy	Soft cheese	1962	+/-	-	+	-
JIM10031	53	France	Milk	1999	+/-	-	+	-
JIM10116	13	Slovakia	Traditional Brinza cheese	2004	+/-	-	+	-
CNRZ385	9	Japan	Sweet yogurt	1971	-	-	-	-
CNRZ703	3	Mongolia	Fermented milk	1974	-	-	-	-
CNRZ759	7	Bulgaria	Yogurt starter	1978	-	-	-	-
CNRZ1066	5	France	Yogurt starter	1986	-	-	-	-
CNRZ1595	17	Austria	Emmental cheese	1979	-	-	-	-
JIM10001	31	France	Cooked pressed cheese	2001	-	-	+	-
JIM10010	33	France	Cooked pressed cheese	2001	-	-	+	+
JIM10020	27	France	Cooked pressed cheese	2001	-	-	-	-
JIM10032	39	France	Milk	1999	-	-	-	-
JIM10037	38	France	Natural starter	2001	-	-	-	-
JIM10050	45	France	Natural starter	2001	-	-	-	-
JIM10055	44	France	Whey	1985	-	-	-	-
JIM10087	51	France	Whey	1984	-	-	-	-
JIM10100	54	France	Milk	2001	-	-	-	-
JIM10114	46	France	Natural starter	1999	-	-	-	-
JIM10117	23	India	Traditional cheese	2004	-	-	-	-
JIM10119	26	India	Traditional cheese	2004	-	-	-	-
LMD-9	8	USA	Cheese	NA	-	-	+	-
LMG18311	6	UK	Yogurt	1974	-	-	-	-
1F8CT	ND	Italy	Curd of grana padano cheese	2012	ND	-	-	-
CNCM I-1360	ND	NA	NA	NA	ND	-	-	-
DGCC7710	ND	NA	Commercial starter	NA	ND	-	+	-
M17PTZA496	ND	Italy	Fontina cheese	1996	ND	+	-	-
MN-ZLW-002	ND	China	Traditionally fermented products	NA	ND	-	-	+
MTCC 5460	ND	India	Fermented milk product (curd)	1984	ND	-	-	+
MTCC 5461	ND	India	Fermented milk product (curd)	1984	ND	-	-	-
MTH17CL396	ND	Italy	Fontina cheese	1996	ND	-	+	+
ND03	ND	China	Commercial starter	NA	ND	-	+	+
TH1435	ND	Italy	Artisanal goat’s milk cheese (milk)	2011	ND	-	+	-
TH1436	ND	Italy	Raw goat milk	2011	ND	-	+	-
TH1477	ND	Italy	Cow milk	2012	ND	+	+	+
TH982	ND	Italy	Buffalo mozzarella curd	2003	ND	-	+	-
TH985	ND	Italy	Buffalo mozzarella whey	2003	ND	-	+	-

^a^, Sequence typing [18, 19];

^b^, (-) poor, (+/-) moderate, (+) strong biofilm production;

^c^, (-) absence and (+) presence of STH8232_0171, STH8232_0714 and STH8232_1361 orthologs;

ND, not determined;

NA, not available.

### Culture and biofilm formation

Planktonic cultures were grown in flasks, without shaking, at 42°C. We assessed *S*. *thermophilus* biofilm formation in a high-throughput system, with crystal violet (CV) staining and a quantitative microplate assay [37]. Each well of a CELLSTAR 96-well cell culture plate (Greiner Bio-one, France), containing 0.2 ml of M17 broth was inoculated with 10 μl of an overnight *S*. *thermophilus* culture and incubated at 42°C for 18 h. We then added 50 μl of 1% CV to each well and incubated the plates at room temperature for 15 min. The wells of the microtiter plates were rinsed three times with 0.2 ml deionized water to remove unattached cells and residual dye. They were then dried. The CV was dissolved in 95% ethanol (1 ml) and its absorbance at 590 nm was determined, to score biofilm formation. We inoculated four quadruplicate wells with each strain for the scoring of biofilm formation and measurement of the optical density of the culture. We performed at least three independent triplicates for each biofilm formation assay.

We investigated the spatial organization of the biofilm for a subset of strains, by growing biofilms in microscopy-grade 96-well plates (Greiner Bio-one, France with a μclear base) and studying them by confocal laser scanning microscopy (CLSM), as previously described [38]. Briefly, surface-associated bacteria were fluorescently labeled in green with 5 μM Syto9 (Invitrogen, France), a cell-permeant nucleic acid marker. The plate was then incubated in the dark for 15 min and mounted on the motorized stage of a Leica SP2 AOBS confocal laser scanning microscope (LEICA Microsystems, France) at the MIMA2 microscopy platform (www.jouy.inra.fr/mima2). All biofilms were scanned at 400 Hz, with a 40× 0.8 N.A. (Leica HCX Apo) water immersion objective lens, and a 488 nm argon laser set at 25% maximum intensity. Syto9 fluorescence was visualized by recording fluorescence in the 500–600 nm range. Three stacks of horizontal plane images (512×512 pixels) with a *z*-step of 1 μm were acquired for each biofilm, from different areas of the well. Three independent experiments were performed for each strain, and the same image acquisition steps were repeated on the same wells after a series of three rinses with water.

Three-dimensional projections of biofilm structures were reconstructed with the Easy 3D function of IMARIS 7.0 software (Bitplane, Switzerland). The quantitative structural parameters of the biostructures, such as biofilm biovolume, substrate coverage and mean thickness, were calculated with PHLIP, a freely available Matlab-based image analysis toolbox (htpp://sourceforge.net/projects/phlip/).

### Cell line culture technique and bacterial adhesion assay

The HT-29 human intestinal cell line (colon adenocarcinoma; ATCC HTB-38) was grown in Roswell Park Memorial Institute medium (RPMI-1640) supplemented with glutamine (2 mM), penicillin (50 U/ml), streptomycin (50 U/ml) and 5% (v/v) FCS (Lonza, Switzerland), at 37°C, under an atmosphere containing 95% and 5% CO_2_. We added 3 × 10^5^ HT-29 cells per well to a 24-well tissue culture plate (TPP, Switzerland), which was then incubated for 24 h. The resulting cell monolayers were washed twice with the cell line culture medium (without antibiotics) before the adhesion assay. Exponentially growing bacteria (OD_600nm_ = 0.9) were collected by centrifugation, washed with phosphate-buffered saline (PBS) and resuspended in RPMI-1640, adjusting the optical density at OD_600nm_ to 0.450. Bacteria (0.5 ml, ~ 10^7^ CFU) were added to the epithelial cell monolayers at a MOI of 50 bacteria per cell, centrifuged for 10 minutes at 4°C and then incubated for 0.5 h at 37°C in an incubator, under an atmosphere containing 5% CO_2_. The monolayers were washed three times with PBS to remove the non-adherent bacteria, and were then lysed with 0.5 ml of sterile 0.5% PBS-Triton X-100. The lysate was vigorously pipetted to release the cell-associated bacteria. Adhesion to polystyrene was assessed as follows. Each well of a CELLSTAR 96-well cell culture plate (Greiner Bio-one, France) was inoculated with 0.2 ml (~ 0.5x10^7^ CFU) of an overnight culture with an adjusted OD_600nm_ of 0.2. The plates were then incubated at 42°C for 1 h. Microtiter plate wells were rinsed three times with 200 μl deionized water to remove the non-adherent cells. The adherent bacteria were recovered by vigorous pipetting in 0.2 ml of trypsin solution (PBS, 25 mM EDTA, 0.25% trypsin [Lonza, Switzerland)]). The serially diluted lysate and bacterial suspension were cultured on M17 agar for the counting of viable bacteria. Adhesion was determined by dividing the number of CFU recovered by the number of CFU for the original inoculum and expressing this ratio as a percentage. HT-29 cell adhesion assays were carried out in duplicate and polystyrene adhesion assays were carried out in triplicate, in at least three independent experiments.

### Isolation of biofilm formation-negative mutants and target determination

*S*. *thermophilus* JIM8232 was mutated by integration of the thermosensitive pGhost9::IS*S1* vector, as previously described [35]. Briefly, cells containing pGhost9::IS*S1* were grown overnight at 30°C in the presence of erythromycin. Stationary-phase cultures were diluted 1:500 in fresh M17 broth without erythromycin, incubated for 150 min at 30°C and shifted to 42°C for 150 min. Samples were then diluted, plated on M17 medium containing erythromycin, and incubated at 42°C. Fifty clones from the 4,000 isolates containing an ISS1-directed insertion reproducibly failed to form biofilms. The pGhost9::IS*S1* insertion site was identified by cloning and sequencing the chromosomal junctions [39]. Junctions were cloned by extracting the chromosomal DNA of mutants, digesting it with *Hin*dIII, and ligating it. We then transformed *E*. *coli* strain TG1repA [40] with selection of erythromycin-resistant clones. Plasmids carrying cloned junctions were extracted from selected clones and the nucleotide sequence of the insert was determined with primers HD1 and HU2. The DNA sequence of the cloned fragment was compared with complete genome sequence of *S*. *thermophilus* JIM8232 [41].

### Construction of mutant strains

A single-crossover insertional mutation of the STH8232_0171, STH8232_0714 and STH8232_1361 loci was constructed with fragments of these genes. For this purpose, an internal fragment of the STH8232_0171, STH8232_0714 and STH8232_1361 genes was amplified by PCR with the primers EG771/EG772, EG758/EG759 and EG762/EG763, respectively. These fragments were inserted into the pGEM-T easy vector (Promega), which was then fused with the integration vector pGhost9 in *Spe*I [39]. The resulting plasmids—pJIM8001, pJIM8002 and pJIM8000—were introduced into *S*. *thermophilus* JIM8232 by electroporation and integrated into the chromosome by single cross-over events, yielding strains JIM9169, JIM9170 and JIM9171, in which the STH8232_0714, STH8232_1361 and STH8232_0171 genes were inactivated, respectively. The SALIVA_0971 locus was deleted from strain JIM8777 and replaced with an Erm^r^ gene. Overlapping PCR was used to prepare donor DNA fragments bounded by the flanking sequences of the deleted material, as previously described [42]. The Erm cassette, derived from pAMβ-1, was amplified from pGhost9 with the EG940/EG941 primers. The upstream and downstream flanking fragments were amplified from strain JIM8777 with the EG1516/EG1517 and EG1518/EG1519 primers, respectively. For assembly, the three fragments were mixed and amplified by overlapping PCR with the EG1516 and EG1519 primers. The PCR product was introduced into the chromosome of strain JIM8777 by XIP-induced transformation [43], with Erm selection, to create strain JIM9395 (JIM8777 ΔSALIVA_0971::*erm*).

### Determination of cell-surface hydrophobicity

Cell surface hydrophobicity was determined by measuring the affinity of cells for hexadecane, as previously described [44]. Briefly, the bacterial cells were harvested by centrifugation (7000 x *g*, 10 min), washed twice and suspended in 150 mM NaCl. The absorbance of the cell suspension at 400 nm was measured and adjusted to 0.8. We then vortexed 2.4 ml of the bacterial suspension thoroughly for 60 s with 0.4 ml hexadecane. The mixture was left at room temperature for 15 min to ensure the complete separation of the two phases before measurement of the absorbance at 400 nm of the aqueous phase (A). The percentage of cells bound to hexadecane was subsequently calculated as: 100 × (0.2/A). Each experiment was performed three times, with two independent bacterial cultures.

### Analysis of the STH8232_0171, STH8232_0714 and STH8232_1361 genes

Comparative genomic analyses were carried out with *S*. *thermophilus* sequences from the strains JIM8232 (FR875178.1) [41], LMD-9 (CP000419.1) [3], ND03 (CP002340.1) [45], LMG18311 (CP000023.1) [21], CNRZ1066 (CP000024.1) [21], MN-ZLW-002 (CP003499.1) [46], CNCN I-1630 (AGFN1000001-376), DGCC7710 (AWVZ01000001-17), MTCC 5460 (ALIK01000001-143), MTCC 5461 (ALIL01000001-144) [47], MTH17CL396 (AZJS01000001-56) [48], M17PTZA496 (AZJT01000001-87) [48], TH1435 (AYSG01000001-39) [49], TH1436 (AYTT01000001-38) [49], TH982 (AZTL01000001-56) [50], TH985 (AZTM01000001-94) [50], TH1477 (AZTJ01000001-64) [50], 1F8CT (AZTK01000001-69) [50], and the sequence of *S*. *salivarius* strain JIM8777 (FR873482.1) [51]. We searched for orthologs of STH8232_0171, STH8232_0714 and STH8232_1361 in the *S*. *thermophilus* strains by PCR amplification, from the genomic DNA of the strains tested, with the EG771/EG772, EG758/EG759 and EG762/EG763 primers, respectively (S1 Table). The genomic region lying between *metK* (STH8232_1369) and *murA1* (STH8232_1353) was amplified by PCR from the genomic DNA of *S*. *thermophilus* strains with the MURA1 and METK primers. The sequences of the STH8232_0714 alleles from *S*. *thermophilus* strains JIM10001, JIM10010, JIM10031, JIM10116 and CNRZ1575 has been deposited in the GenBank database under accession numbers KF717044, KF717043, KF717042, KF717041, and KF717045, respectively.

### Statistical analysis

GraphPad Prism version 6 (GraphPad software Inc., La Jolla, CA, USA) was used for statistical analyses. A *P* value < 0.05 was considered to be statistically significant.

## Results

### *Streptococcus thermophilus* is a poor biofilm producer

**Fig 1:**
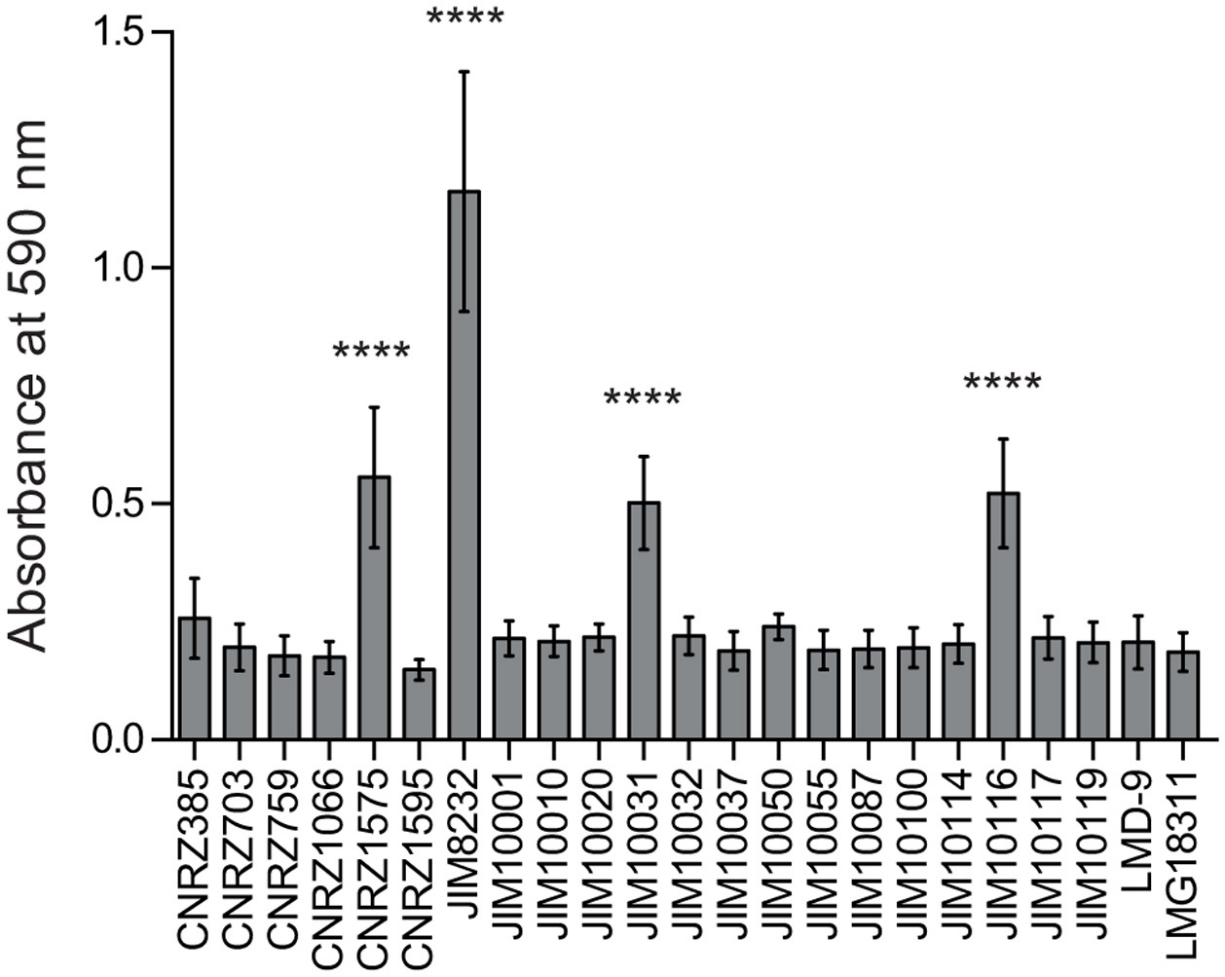
Differences in biofilm formation between natural *S*. *thermophilus* isolates. Biofilm formation was evaluated by quantitative microtiter plate assays based on the staining of 18-hour-old biofilms with crystal violet (CV). The columns show the mean values for CV absorbance (at 590 nm) of at least four independent experiments performed in duplicate, and the error bars indicate standard deviations. Statistical analyses included one-way ANOVA and the Bonferroni post hoc test, comparing the mean value for each strain with that of the strain with the lowest mean value (CNRZ1595). Asterisks denote statistically significant differences (*P* < 0.0001).

We investigated the ability of a representative collection of 23 *S*. *thermophilus* isolates to form biofilms. This collection contained isolates obtained over a long time period (1962 to 2004) from diverse products (including cheeses, yogurts, fermented milks and starters), different geographic locations (10 countries) and belonging to different MLST lineages (Table 1). We used a static monospecies biofilm model in which the cohesive mature biofilm was measured in a polystyrene 96-well microplate after crystal violet (CV) staining (Fig 1). All strains had similar growth rates in planktonic cultures (S1 Fig). However, only four produced relevant biofilms, with strain JIM8232 producing significantly more biofilm than any of the other strains.

**Fig 2:**
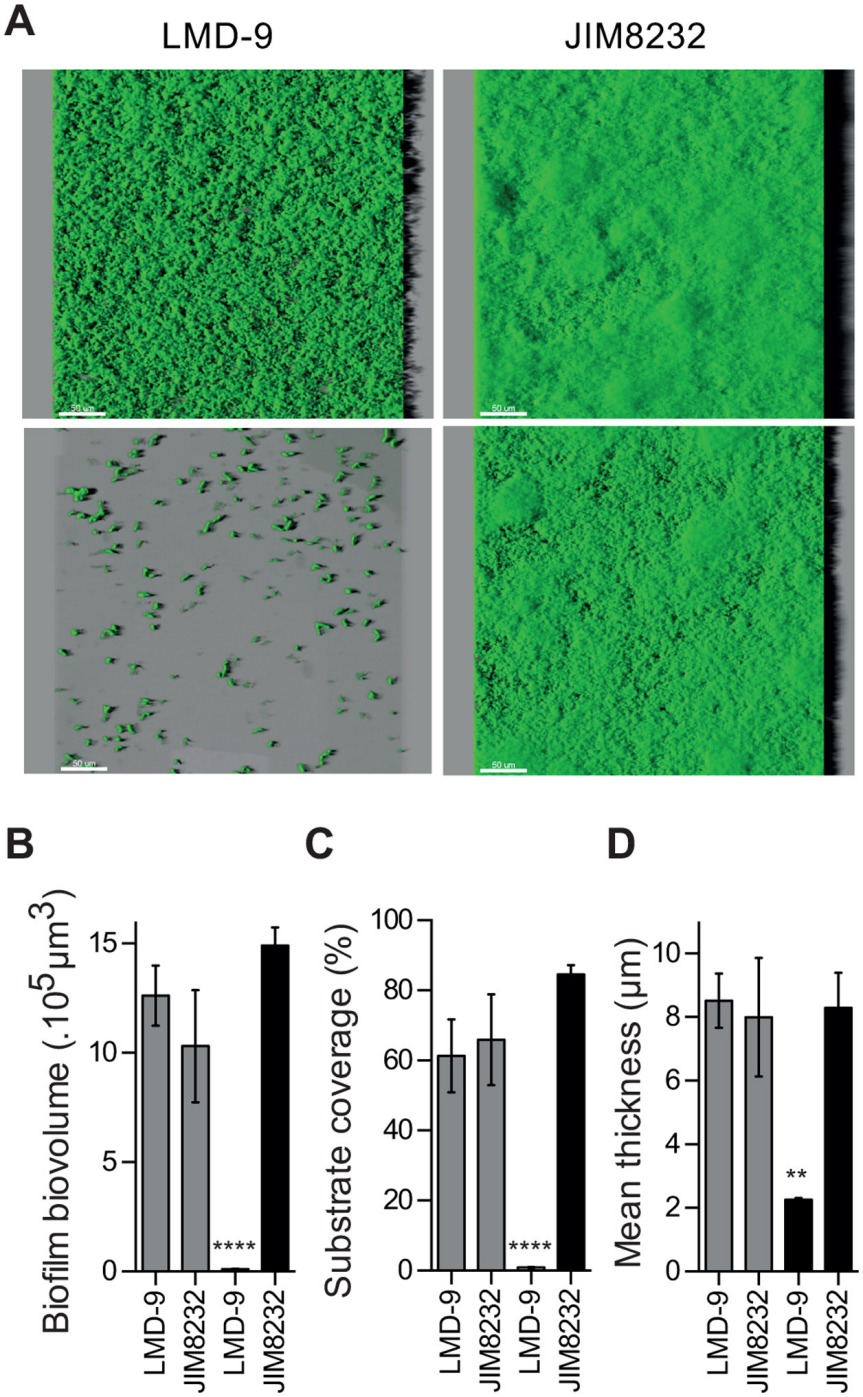
Spatial organization of *S*. *thermophilus* LMD-9 and JIM8232 biofilms, from series of confocal images. (A) Three-dimensional confocal projection of 18-hour-old biofilms produced by strains LMD-9 and JIM8232, before (upper panel) and after (lower panel) washing, obtained from *xyz* confocal image series with IMARIS software. The images show a representative and aerial view of biofilm structures, with the virtual shadow projection on the right. Scale bars correspond to 50 nm. (B) Mean biofilm biovolume, (C), substrate coverage, and (D) biofilm thickness of the 18-hour-old biofilms produced by strains LMD-9 and JIM8232 before (gray bars) and after (black bars) washing, extracted from confocal images with the PHLIP Matlab tool. The columns show the means of three independent experiments performed in triplicate, and the error bars indicate the standard deviation. Statistical analysis included Student’s paired *t*-tests (**** *P* < 0.0001, **, *P* < 0.01 for comparisons with JIM8232 after washing).

For validation of the CV assay and assessment of the spatial organization of the biofilms, we analyzed the biofilms formed by JIM8232 and LMD-9, used as a negative control, by CLSM. Representative 3D projections of the biofilm formed were obtained, before and after the washing steps used to remove loosely attached and sedimented bacteria (Fig 2A upper and lower panels, respectively). These images were used for quantitative measurements of biofilm structure, including biovolume, substrate coverage and mean thickness (Fig 2B–2D). No significant differences were found between the biofilms formed by strains LMD-9 and JIM8232 before washing. However, all structural measurements were significantly lower after washing for strain LMD-9 (*P* < 0.0001), whereas washing had no effect on the structural measurements for JIM8232 (*P* > 0.05). CLSM images revealed that the cells in the washed LMD-9 biofilm formed very small microcolonies on the polystyrene surface, corresponding mostly to surface-associated isolated cell chains (Fig 2A, lower panel). By contrast, the biofilm-producing strain JIM8232 formed a cohesive, washing-resistant film of cells on the surface of the substrate. The JIM8232 biofilm had a relatively homogeneous structure and it covered the entire available surface. These observations confirm that strains JIM8232 and LMD-9 may be considered to be strong and poor biofilm producers, respectively. As most *S*. *thermophilus* strains behave like strain LMD-9, at least under our experimental conditions, this species can be classified as a poor biofilm producer.

### The poor biofilm-formation ability of *Streptococcus thermophilus* is correlated with a low capacity for adhesion

**Fig 3:**
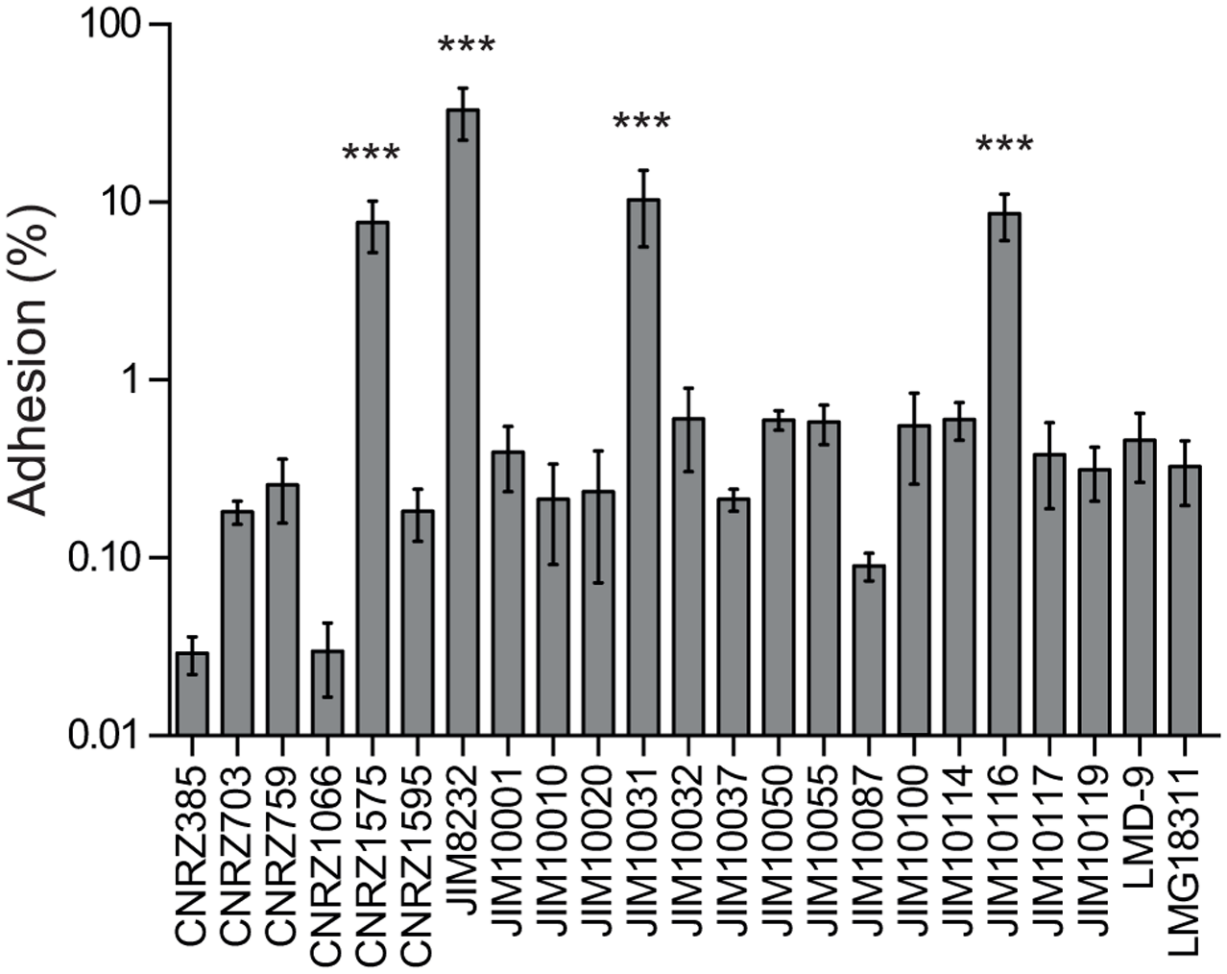
Initial adhesion of natural *S*. *thermophilus* isolates to polystyrene microplates. The numbers of adherent bacteria were determined as colony-forming units on M17 solid agar plates and are expressed as percentages of the input inoculum. The columns show the mean percentages of adherent bacteria from at least three independent experiments performed in duplicate, and the error bars indicate standard deviations. Statistical analyses included one-way ANOVA and Bonferroni post hoc tests for comparisons of the mean value for each strain with that of the strain with the lowest mean value (CNRZ385). Asterisks denote statistically significant differences (*P* < 0.001).

Biofilm development is a complex process with multiple steps following the attachment of the microbe to a surface [52]. We investigated whether the failure of most of the *S*. *thermophilus* strains tested to form biofilms was due to an inability to adhere to the substrate. We counted the number of bacterial cells adhering to polystyrene after one hour of incubation in the wells of a microtiter plate (Fig 3). The ability of strains to form biofilms was correlated with the percentage of cells attaching to polystyrene (*r* ^*2*^ = 0.9769, *P* < 0.0001, Pearson’s correlation). The strains could be classified as strong, moderate and poor biofilm producers, and these classifications corresponded to strains with strong (33%), intermediate (from 7.8 to 11%) and weak (from 0.013 to 0.63%) adhesion, respectively. Our findings thus suggest that the poor ability of most *S*. *thermophilus* strains to form biofilms is a consequence of their poor initial adhesion to surfaces.

### Identification of genes involved in the formation of biofilms by strain JIM8232

We used the pGhost9::IS*S1* vector [39] to generate a transposon mutant library for identification of the genetic factors involved in biofilm formation. We screened over 4,000 insertion mutants for their ability to form biofilms in 96-well plate CV assays. Fifty mutants reproducibly failed to form biofilms. The chromosomal DNA fragments flanking the pGhost9::IS*S1* insertion site of 23 of these mutants were rescued in *E*. *coli*, sequenced and compared with the full genome sequence of JIM8232 [41]. The functions of the genes identified by this procedure were analyzed and we searched for homologs in the publicly available *S*. *thermophilus* genome sequences. The transposons in these mutants had affected eight different genes (S2 Table).

Two of them, STH8232_1361 and STH8232_0714, were each inactivated by several independent transposition events, i.e. the transposon was found at different positions within these genes in different insertion mutants (14 mutants for STH8232_1361, 3 mutants for STH8232_0714), which had lost their biofilm formation capability. This observation constitutes a strong indication for their potentially critical role in biofilm formation and these genes were therefore selected for further study. STH8232_1361 and STH8232_0714 encode a transmembrane protein from the polysaccharide transporter (PST) family (TCDB database) and a cell surface-exposed protein with a MucBP domain (MUCin-Binding-Protein, Entry PF06458), respectively. STH8232_0171 was also selected for further study because this gene is present in only 3 of 18 currently sequenced *S*. *thermophilus* strains and the one available for testing, JIM8232, formed biofilms. It encodes a predicted cytoplasmic protein (Psort prediction) with unknown function and containing a NACHT domain (nucleoside-triphosphatase domain, Entry PF05729). The remaining five genes were not studied further because they were targeted only once by the transposon and they are present in most sequenced *S*. *thermophilus* strains, including those unable to form biofilms (S2 Table).

**Fig 4:**
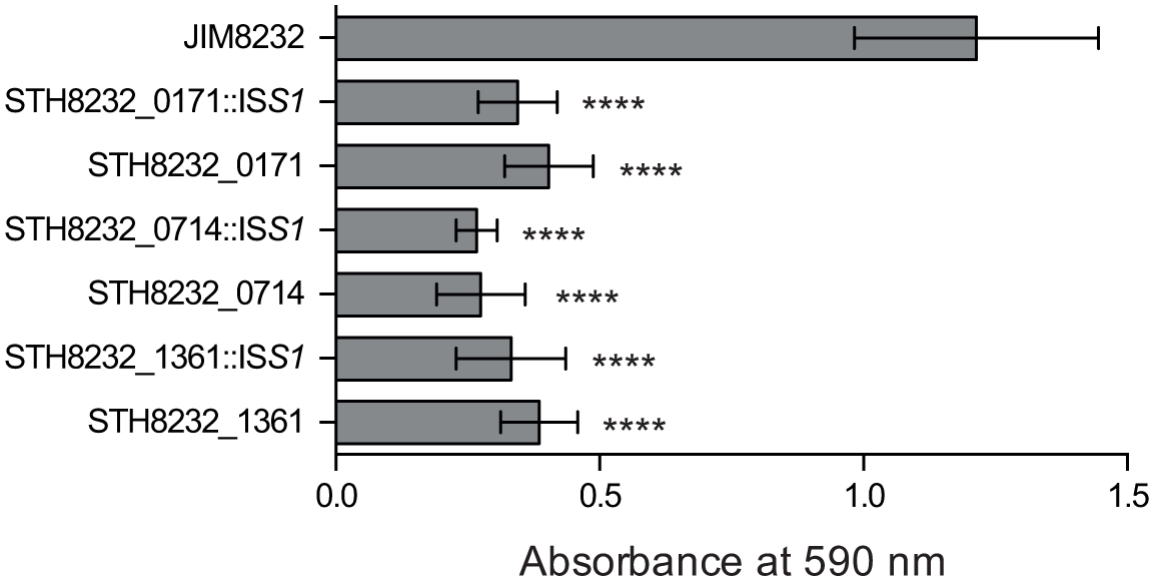
Biofilm formation by *S*. *thermophilus* JIM8232 and its isogenic mutants. The biomass of 18-hour-old biofilms from the IS*S1* transposon mutants (STH8232_0171::IS*S1*, STH8232_0714::IS*S1*, STH8232_1361::IS*S1*) and the corresponding reconstructed mutants (STH8232_0171 [JIM9171], STH8232_0714 [JIM9169], STH8232_1361 [JIM9170]) was measured in a crystal violet (CV) assay. The columns show mean CV absorbance values for at least four independent experiments performed in triplicate, and the error bars indicate standard deviations. The significance of the differences between JIM8232 and its isogenic mutants was determined in Student’s paired *t*-tests (****, *P* < 0.0001).

For independent confirmation of the involvement of STH8232_1361, STH8232_0714 and STH8232_0171 in JIM8232 biofilm formation, and to exclude the possibility of secondary mutations, we inactivated these genes in the wild-type strain and evaluated the biofilm-forming ability of the corresponding knockout mutants (Fig 4). The biofilm-forming capability of each of the three mutants was similar to that of the corresponding IS*S1* transposon mutants and was significantly lower (approximately 70%; *P* < 0.0001) than that of the wild-type strain confirming that these three genes directly contribute to biofilm formation. We further analyzed the corresponding genomic regions in sequenced *S*. *thermophilus* strains and, using a PCR-based approach, in the 23 strains of our collection, to determine the functions and variability of these genes.

### STH8232_1361 is responsible for a remnant function involved in surface polysaccharide production

**Fig 5:**
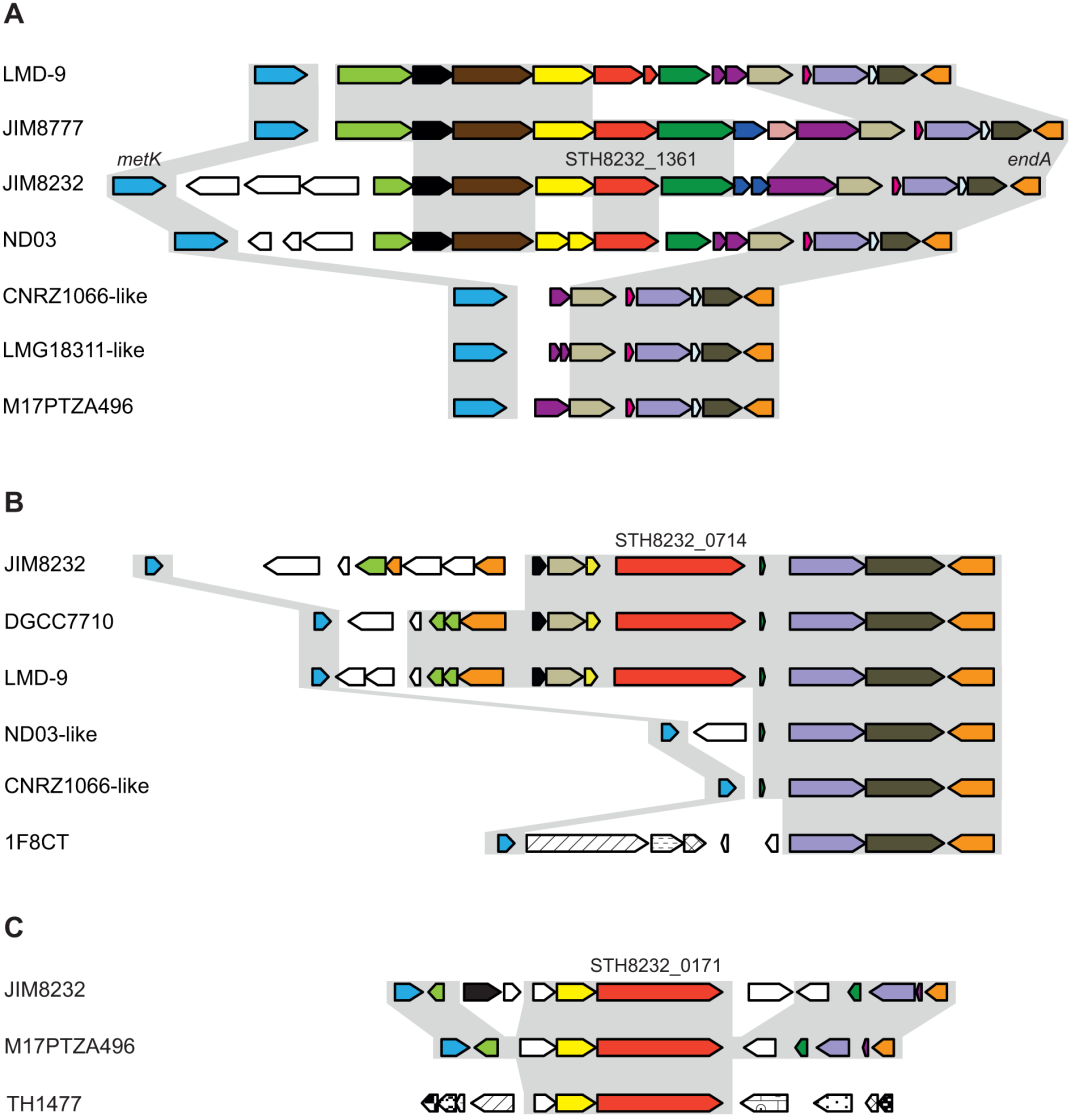
Schematic diagram of the STH8232_1361, STH8232_0714 and STH8232_0171 loci. (A) The STH8232_1361 region *in S*. *thermophilus* LMD-9, JIM8232, ND03, CNRZ1066-like (CNRZ1066, TH985, TH982, TH1436, DGCC7710, 1F8CT), LMG18311, M17PTZA496-like (M17PTZA496, TH1435) and *S*. *salivarius* JIM8777. (B) The STH8232_0714 region in *S*. *thermophilus* JIM8232, DGCC7710, LMD-9, ND03-like (ND03, MN_ZLW_002), CNRZ1066-like (CNRZ1066, LMG18311) and 1F8CT. (C) The STH8232_0171 region in *S*. *thermophilus* JIM8232, M17PTZA496 and TH1477. The orthologous genes in each panel are represented by pentagons of the same color, except for mobile elements, which are shown in white. The lengths of genes and intergenic regions are drawn to scale.

The STH8232_1361 gene is part of a large cluster of 16 open-reading frames (ORFs) flanked upstream by *metK* (STH8232_1369) and downstream by *endA* (STH8232_1351) (Fig 5A). The STH8232_1361 locus appears to be variable in *S*. *thermophilus*. A complete or usable draft genome sequence is available for 12 *S*. *thermophilus* strains. The STH8232_1361 region (from STH8232_1368 to STH8232_1356) is entirely absent from nine of these strains, including LMG18311 and CNRZ1066, and contains numerous pseudogenes and transposases in the others (Fig 5A). Conversely, a similar, intact region is found in the closely related species *S*. *salivarius* JIM8777. Fifteen of our 23 *S*. *thermophilus* strains yielded a PCR product corresponding to the *metK-murA1* region and of similar size to that from CNRZ1066 and LMG18311 (~3.5 kb). These strains thus contained a cluster with a large deletion encompassing STH8232_1361 (Fig 5A and S2 Fig). A PCR product with a size corresponding to that of STH8232_1361 was obtained only from LMD-9 (but as a pseudogene), JIM8232 and JIM10010, confirming the absence of this gene from most of the *S*. *thermophilus* strains studied (Table 1). These findings indicate that, in most *S*. *thermophilus* strains, the STH8232_1361-containing locus has undergone multiple inactivation events, some of which have resulted in gene loss, consistent with the regressive evolution occurring in this species [21–23].

This cluster contains genes encoding functions associated with extracellular activities, including polysaccharide synthesis in particular: STH8232_1361 (polysaccharide transporter), STH8232_1362 (glycosyltransferase I), STH8232_1355 (UDP-glucose 4-epimerase), and STH8232_1353 (UDP-N-acetylglucosamine 1-carboxyvinyltransferase). A search of complete genomes revealed the presence of homologs of STH8232_1361 in several phyla. In particular, homologs of STH8232_1361 and STH8232_1362, which are always organized in tandem in all STH8232_1361-containing bacteria, were found to be prevalent in host-associated bacterial families, including many Streptococcaceae, Ruminococcaceae, Bifidobacteriaceae, Clostridiaceae, Coriobacteriaceae, Lachnospiraceae and Paenibacillaceae. All these families contain bacteria from the intestinal flora. The homologs identified include *PelG* and *PelF* from *Pseudomonas aeruginosa*; these genes encode proteins involved in the synthesis of the carbohydrate-rich polymers playing a key role in initial interaction of the bacterium with solid surfaces and biofilm structure [53–55]. Thus, the STH8232_1361 locus in JIM8232 is involved in the production of surface polysaccharides likely to play a role in biofilm formation, and has been lost from other *S*. *thermophilus* strains.

### STH8232_0714 encodes a surface-exposed protein and is part of a locus that has been subject to gene loss

Half the sequenced *S*. *thermophilus* genomes contain an ortholog of STH8232_0714 (S2 Table), and this gene was detected by PCR amplification in seven of the 23 strains we tested for biofilm formation (Table 1). The STH8232_0714 genomic region was compared between eight sequenced strains (Fig 5B): only two (LMD-9 and DGCC7710) contained a gene orthologous to STH8232_0714. In strain JIM8232, STH8232_0714 is followed by an ABC transporter and preceded by several genes and mobile elements. The transporter is conserved in the genomes of the other seven strains, but the upstream region is highly variable. The STH8232_0714 locus thus presents features of reductive evolution, including gene inactivation and almost total deletion.

A remarkable feature of STH8232_0714 and its ortholog in LMD-9 is their substantial divergence (91% identity), greater than the average for all the genomes considered (99.8% identity, [21, 22]). The divergent nucleotides are clustered in an ~1 kb inner region (positions 717 to 1856, displaying 15% nucleotide divergence, and 19% divergence at the amino-acid level), whereas the flanking regions contain only two mismatches. The sequence of this variable region was determined in the other five strains and compared with the sequences of the LMD-9 and JIM8232 orthologs (S3 Fig). The sequences clustered into two well separated groups containing the JIM8232 and LMD-9 orthologs, respectively. However, these two groups each contained biofilm producers and non-producers. The variability of STH8232_0714 cannot therefore be considered to be directly related to biofilm formation.

STH8232_0714, which is flanked by a promoter and terminator sequence, is predicted to encode a cell surface protein containing a MucBP domain and belonging to a group of adhesins facilitating the attachment of bacteria to host cells. MucBP-containing proteins have been found in more than 315 bacterial species (Pfam analysis with PF06458 as the input, http://pfam.xfam.org), mostly from the Streptococcaceae (211 species) and Lactobacillaceae (86 species), many found in association with animal mucosa. The putative protein encoded by STH8232_0714 thus has several features of proteins involved in adhesion to surfaces. This gene is highly polymorphic and is subject to gene loss in *S*. *thermophilus*.

### STH8232_0171 was acquired by HGT

The STH8232_0171 gene, which is flanked by a promoter and terminator sequence, is surrounded by ORFs encoding transposases and maps to a 40-kb genomic island encoding proteins involved in the synthesis of a yellow pigment—an atypical trait of this species and of streptococci in general [41]. Moreover, its GC% (29.1%) differs significantly from the mean value for the JIM8232 chromosome (38.9%). This gene was also present in the genomes of *S*. *thermophilus* strains M17PTZA496 and TH1477 (100% identity), which contain part of the JIM8232 40-kb genomic island. PCR-based tests showed this gene to be absent from the 23 strains studied (Table 1 and S2 Table). BLAST searches of the genomes of other species for the STH8232_0171 gene product gave significant matches to putative proteins from *Enterococcus cecorum* DSM 20682 (79%), *S*. *oralis* SK255 (78%), and *S*. *mitis* SK95 (78%). In *E*. *cecorum*, the corresponding gene is located in a genomic island flanked by an integrase and IS elements, whereas, in *S*. *oralis* and *S*. *mitis*, it is present in a variable region. These various findings indicate that the STH8232_0171 gene was acquired by HGT.

### Role of STH8232_0171, STH8232_0714 and STH8232_1361 in biofilm formation

**Fig 6:**
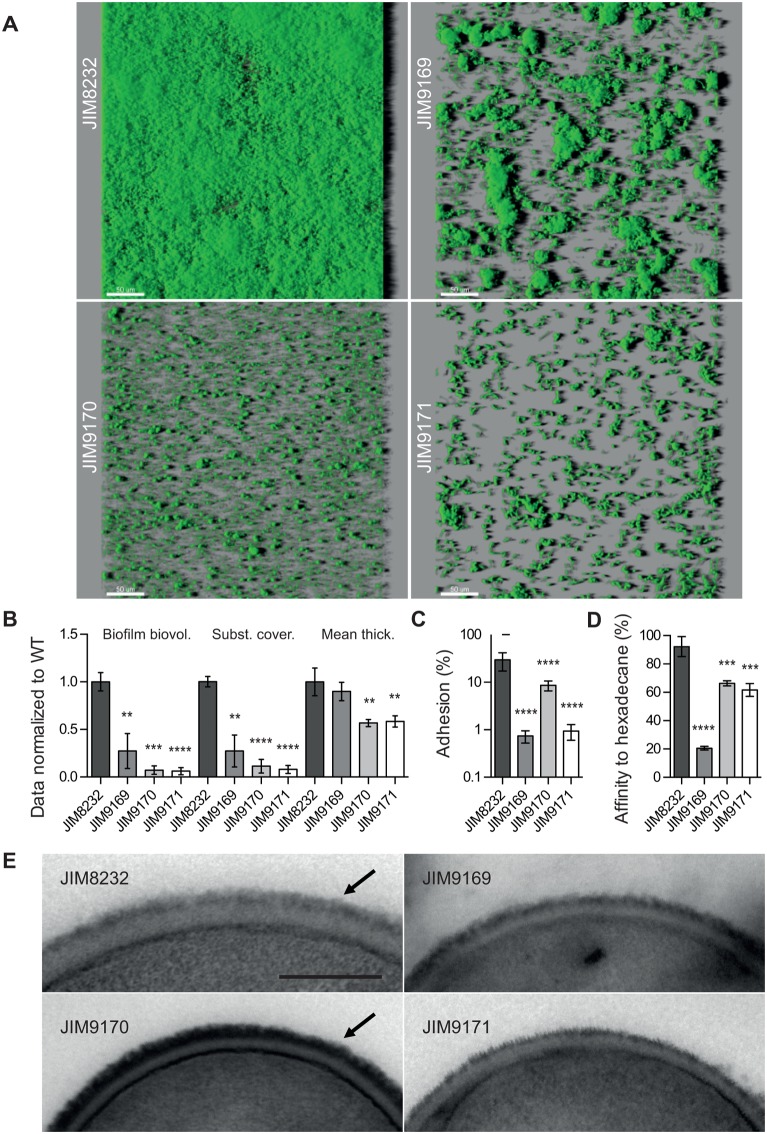
Role of STH8232_0171, STH8232_0714 and STH8232_1361 in biofilm formation. (A-B), Properties of biofilms formed by *S*. *thermophilus* JIM8232 and its isogenic mutants JIM9169 (STH8232_0714), JIM9170 (STH8232_1361) and JIM9171 (STH8232_0171). Panel (A) shows 3-D reconstructions of representative confocal images of biofilms and panel (B) shows biofilm biovolumes (Biofilm biovol.), substrate coverages (Subst. cover.) and mean thicknesses (Mean thick.), normalized against those for JIM8232, for each strain, as measured in nine different CLSM image stacks from three independent experiments. Scale bar, 50 μm. (C), Bacterial adhesion (after 1 h) to polystyrene wells. Adherent bacteria were counted as colony-forming units on M17 solid agar plates and the results are expressed as percentages of the input inoculum. The columns show the mean values for five independent experiments, each performed in triplicate, and the error bars indicate the standard deviations. (D), Percentage of bacteria bound to hexadecane solvent. The columns show the mean values for four independent experiments performed in duplicate, and the error bars indicate standard deviations. (E), Scanning electron microscopy of *S*. *thermophilus* JIM8232 and its isogenic mutants. Arrows indicate the cell wall polysaccharide layer. Scale bar, 0.2 μm. The significance of the differences between JIM8232 and its isogenic mutants was determined in Student’s paired *t*-tests (****, *P* < 0.0001; ***, *P* < 0.001; **, *P* < 0.01).

We investigated the role of these three genes in biofilm formation, by analyzing the biofilms formed by JIM8232 and the three mutants by CLSM (Fig 6A and 6B). The mutations led to different three-dimensional structural abnormalities, indicating different roles for the corresponding proteins in biofilm development. The STH8232_0714 mutant (JIM9169) formed relatively independent large cell clusters embedded in an apparently smooth matrix that did not cover the entire surface of the polystyrene. STH8232_0714 may therefore be involved in cell-substratum interactions and, in particular, the initial adhesion occurring at the start of biofilm development. The STH8232_0171 mutant (JIM9171) presented a similar pattern, but with smaller clusters of cells. STH8232_0171 may therefore be involved in both primary adhesion and biofilm cohesion, through cell-cell and/or cell-matrix interactions. The STH8232_1361 mutant (JIM9170) cells displayed even higher levels of adhesion to the substratum than the other two mutants and it formed no clusters. This suggests that STH8232_1361 is principally involved in biofilm cohesion. These observations indicate that STH8232_0714, STH8232_1361 and STH8232_0171 play different roles (initial adhesion and biofilm cohesion) in the early stages of biofilm development.

We then studied the cell surface-related properties—such as adhesion to a polystyrene surface, hydrophobicity and cell morphology—of the STH8232_0171, STH8232_0714 and STH8232_1361 0171 mutants. The number of cells adhering to the polystyrene surface was much smaller for the STH8232_0714 and STH8232_0171 mutants (JIM9169 and JIM9171, respectively) than for the wild-type (approximately 1%, *P* < 0.0001); the number of adherent cells was intermediate for the STH8232_1361 mutant (JIM9170; 29% of the wild-type value, *P* < 0.0001; Fig 6C). These results were consistent with the CLSM analysis and confirmed the requirement of STH8232_0171 and STH8232_0714 for surface attachment during the early stages of biofilm formation. They indicate that STH8282_1361 is involved in interactions of cells with the substrate, but to a lesser extent. Differences in hydrophobicity between the wild-type and mutant cells were estimated in MATH tests. JIM8232 displayed a high affinity for the solvent (> 90%, Fig 6D), reflecting its hydrophobic nature. All the mutant strains had significantly lower solvent affinities. However, the affinity for hexadecane of the STH8232_0171 and STH8232_1361 mutants was only moderately lower (61% to 68%), whereas that of the STH8232_0714 mutant was substantially lower and similar to that of hydrophilic strains. STH8232_0714 is therefore likely to be a cell surface protein determining the hydrophobicity of JIM8232 cells, possibly facilitating hydrophobic interactions with the substrate during adhesion. Transmission electron microscopy showed that strains JIM9169, JIM9171 and JIM8232 were indistinguishable in terms of their overall morphologies (data not shown). However, the external layer corresponding to cell wall polysaccharides was denser in the STH8232_1361 mutant than in the other strains (Fig 6E), consistent with a role for STH8232_1361 in polysaccharide synthesis.

### The biofilm-associated genes of JIM8232 are involved in adhesion to epithelial cells

**Fig 7:**
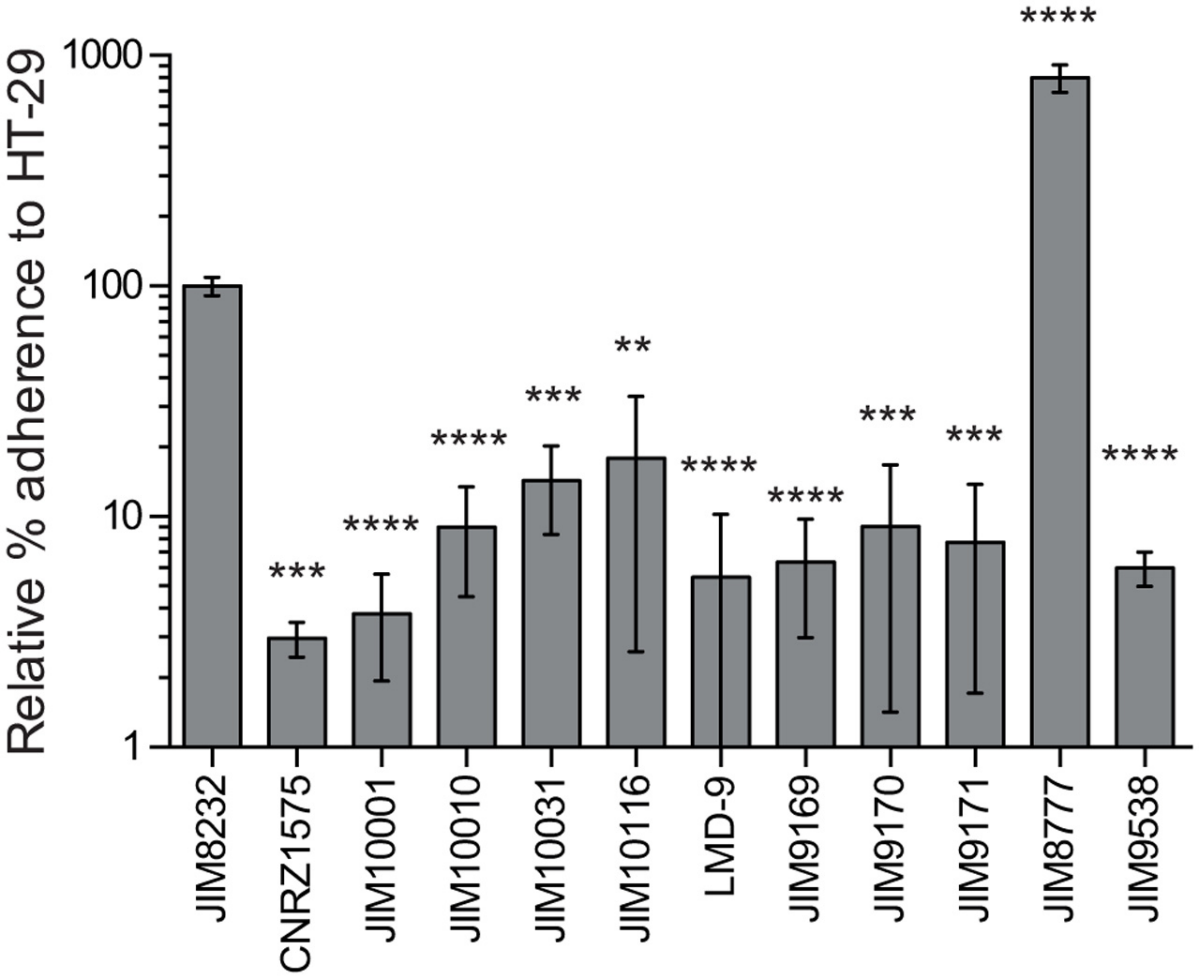
Adhesion to HT-29 cells of several *S*. *thermophilus* and *S*. *salivarius* strains. Adherent bacteria were counted as colony-forming units on M17 solid agar plates and the results are expressed as percentages of the input inoculum. The columns show the mean values for three independent experiments, each performed in triplicate, and the error bars indicate the standard deviations. Student’s paired *t*-test was used for statistical analysis (**** *P* < 0.0001 for comparisons with JIM8232 or JIM8777).

STH8232_0714 and STH8232_1361 display sequence similarity to genes present in many host-associated bacteria but absent from most *S*. *thermophilus* strains (see above). These genes may, therefore, be remnants from the ancestor of *S*. *thermophilus*, believed to be closely related to the *S*. *salivarius* and *S*. *vetibularis* commensals. This suggests a role for these genes in interactions between the bacterium and its host. We investigated this possibility, by evaluating the adhesion of wild-type and mutant *S*. *thermophilus* and *S*. *salivarius* strains to HT-29 epithelial cells (Fig 7). JIM8232 was the natural isolate of *S*. *thermophilus* displaying the strongest adhesion to HT-29 cells (*P* < 0.05), although its binding affinity was only one tenth that of the *S*. *salivarius* commensal strain JIM8777. The binding of STH8232_0171, STH8232_0714 and STH8232_1361 mutants was only 10% that of JIM8232, implicating these genes in adhesion to HT-29 cells. A mutant for the *S*. *salivarius* STH8232_1361 ortholog (JIM9395) also bound significantly less strongly than the control to HT-29 cells, confirming the involvement of this gene in host cell interaction. Thus, unlike most *S*. *thermophilus* strains, JIM8232 can adhere to epithelial cells via a mechanism similar to that of one of its commensal relatives, *S*. *salivarius*.

## Discussion

In this study, we explored the biofilm-forming ability of 23 representative *S*. *thermophilus* isolates of diverse origins. We found that this species was a poor producer of biofilms, and that this property was associated with a lack of ability to adhere firmly to surfaces, the first step in biofilm development [52]. However, one strain, JIM8232, was a strong biofilm producer. Screening for JIM8232 mutants with impaired biofilm formation led to the identification of three genes—STH8232_0714, STH8232_1361 and STH8232_0171—required for biofilm production by *S*. *thermophilus*. Several observations provide compelling evidence that the biofilm negative phenotype of the transposon insertion mutants is the result of the interruption of these genes rather than the result of secondary mutations or polar effects on downstream genes. Firstly, reconstruction of mutations for these genes in the wild-type strain rendered this strain biofilm negative. Secondly, both STH8232_0171 and STH8232_0714 are followed by putative rho-independent terminators and promoter regions indicating that transposon insertion should not significantly affect downstream gene expression. Lastly, the fact that 14 independent transposon insertions targeted STH8232_1361 and 3 STH8232_0714, but none the downstream genes indicates that their inactivation is specifically required to produce a biofilm negative phenotype. Biofilm development is a dynamic, complex multistep process involving the initial attachment of planktonic bacterial cells to a surface, production of the extracellular matrix, cluster formation and development and maturation of the biofilm architecture [52]. We show that the functions encoded by STH8232_0714, STH8232_1361 and STH8232_0171, which are absent from most *S*. *thermophilus* strains (Table 1), are related to early stages of biofilm development and are therefore crucial for biofilm formation. The surface-exposed STH8232_0714 protein may facilitate hydrophobic interactions with the substrate during primary adhesion, and the STH8232_1361-dependent polysaccharide matrix may trigger adhesion and biofilm cohesion [56]. Phenotypic analysis of the STH8232_0171 mutant indicated that it was also involved in these two steps of biofilm formation. However, the product of this gene is predicted to be cytoplasmic, and its precise role in adhesion and biofilm cohesion therefore remains unclear. The deduced amino-acid sequence of the STH8232_0171 gene product contains a domain predicted to be involved in signal transduction mechanisms, suggesting a possible role in the regulation of biofilm formation.

The study of these three loci provides information about their evolutionary history. STH8232_0714 and STH8232_1361 are located within gene clusters presenting features of reductive evolution: in most *S*. *thermophilus* strains, there is evidence to suggest that major rearrangements to these clusters have occurred, including their almost total deletion. Similar clusters are present in the closely related commensal streptococci, from which *S*. *thermophilus* recently emerged as a food bacterium. In commensal bacteria, the functions associated with the homologs of STH8232_0714 and STH8232_1361 relate to adhesion and interaction with the host mucosa (this work) [57–60], which may lead to biofilm formation, an important feature frequently associated with colonization capability [61–63]. Such functions are probably dispensable for growth in milk and loss of the functions of the STH8232_0714 homologs has been reported in other domesticated bacteria [23, 57, 64]. The STH8232_0714- and STH8232_1361-dependent biofilm phenotype thus appears to be an ancestral trait that, like other features, has been lost during the genetic domestication of *S*. *thermophilus* [21–23]. An analysis of STH8232_0171, the other biofilm-associated gene identified in this study, revealed a different story. STH8232_0171 was found in only three strains, all isolated from raw milk. In JIM8232, it maps to a 40-kb island also involved in the synthesis of a yellow pigment not typically present in this species or other streptococci [41]. Its close homologs in commensal bacteria are also carried by genomic islands, suggesting that this gene may be exchanged through horizontal gene transfer. The function of this gene in these bacteria is unknown, but our findings indicate that it is associated with biofilm formation in *S*. *thermophilus* JIM8232. The biofilm formation phenotype displayed by JIM8232 therefore seems to be dependent on both the inheritance of ancestral functions and the acquisition of another gene, all related to the commensal lifestyle.

The poor biofilm production capacity of *S*. *thermophilus* is intriguing. Association with a biofilm is the predominant lifestyle in bacteria and a key strategy for survival in harsh environments. Many *S*. *thermophilus* strains have been isolated from equipment bearing multispecies biofilms, such as wood vats, used for the fermentation of traditional products [16, 32, 33]. Such biofilms may act as a reservoir for this species. Strain JIM10116 was isolated from traditional Brinza cheese. It has a moderate ability to form biofilms and may be representative of such strains. The development of multispecies biofilms, which probably predominate in natural conditions, results from cooperation and interactions between different microbial species [27, 28, 65, 66]. Our biofilm model, involving the attachment of a single strain to an abiotic surface, may therefore not be representative of the natural environment of *S*. *thermophilus*.

A biofilm may not be required to ensure the resilience of this bacterium in many products. In yogurt, the fermentation process is initiated by the addition of the preceding yogurt culture, whereas, in several traditional thermophilic dairy production processes, the acidification step is started by back-slopping. Moreover, current fermentation processes are based on the inoculation of milk with commercial starters selected on the basis of criteria such as rapid growth in fermenters. Biofilm producers may not be the most appropriate strains for these conditions. The propagation of commercial strains as planktonic cultures adapted to industrial processes may have led to the loss of genetic material, as demonstrated for natural isolates cultured under laboratory conditions [67–69]. Consequently, genetic determinants, such as those involved in bacterium-host interactions identified in this study, may have been lost spontaneously or even eliminated by counter-selection in most food-associated *S*. *thermophilus* lineages. The 23 strains included in this study are representative of such strains because most are domesticated strains with a long history of use in dairy fermentations. JIM8232, a pigment producer isolated from a milk tank truck, may not therefore be representative of strains selected for dairy fermentation.

Our study raises questions about the origins of such strains. The JIM8232 genome contains functional clusters that are probably vestiges of the commensal origin of the *S*. *thermophilus* ancestor. It also contains islands, such as that carrying STH8232_0171, another feature consistent with a commensal origin. This strain has the PrtS island allowing casein assimilation, which was probably acquired by transfer from an animal-associated streptococcal strain [19]. Such repeated exchanges of genetic material between *S*. *thermophilus* and bacteria not thought to occupy the same ecological niche raise questions about how such transfers occur. One possibility is that *S*. *thermophilus* occupies an as yet undiscovered animal-associated niche on farms. The ability of JIM8232 to adhere to epithelial cells, and the isolation of *S*. *thermophilus* from raw milk and cow udders are consistent with this possibility. However, the presence of such strains in animals has yet to be confirmed. Alternatively, such transfers may be facilitated by the frequent addition of *S*. *thermophilus* to bacterial mixtures used as probiotics for animals, including pigs and chickens, or as starters for silage. This extensive release of *S*. *thermophilus* in the farm environment would probably facilitated horizontal gene transfer events between this species and the animal gut microbiota, as proposed for antibiotic resistance genes [70, 71]. The resulting strains could then contaminate milk, leading to their dissemination throughout the food chain. The selection of *S*. *thermophilus* strains with functional attributes other than those required for milk fermentation, and their use for new applications, such as animal probiotics, could therefore contribute to the emergence of new strains and the evolution of the *S*. *thermophilus* genome. *S*. *thermophilus* is currently considered to be innocuous and is consumed daily in massive amounts around the world with no known harmful effects on human health. However, given the ability of this species to acquire new genes readily and its extended use at sites other than dairies, particular attention should be paid to the possibility of undesirable traits disseminating among *S*. *thermophilus* starter strains.

## Supporting Information

S1 Fig Final cell density of *S*. *thermophilus* natural isolates grown in microtiter plates. Columns represent means absorbance at 600 nm of at least four independent experiments performed in duplicate, and bar errors indicate standard deviations.
(TIFF)

S2 Fig STH8232_1361 containing locus among *S*. *thermophilus* natural isolates. The genomic region comprised between metK (STH8232_1369) and murA1 (STH8232_1353) was amplified by PCR from genomic DNA of S. thermophilus strains with the MURA1 and METK primers. Numbers on the top of the electrophoresis gel lanes referred to the name strains as following: M, marker; 1, LMD-9; 2, JIM8232; 3, JIM10010; 4, JIM10116; 5, JIM10032; 6, JIM10119; 7, JIM10001; 8, JIM10020; 9, JIM10031; 10, JIM10037; 11, JIM10050; 12, JIM10055; 13, JIM10087; 14, JIM10100; 15, JIM10114; 16, JIM10117; 17, LMG18311; 18, CNRZ1066; 19, CNRZ759; 20, CNRZ1575; 21, CNRZ1595.
(TIFF)

S3 Fig Phylogenic tree of STH8232_0714 orthologous genes in *S*. *thermophilus*. Blue, moderate and strong biofilm producing strains; Red, no or poor biofilm producing strains; Black, strains for which biofilm formation ability was not determined.
(TIFF)

S1 Table Bacterial strains, plasmids and primers used in this study. (TIFF)

S2 Table Features of genes identified as involved in biofilm formation of strain JIM8232 by a genome-wide mutagenesis approach and their occurrence with percent identity among sequenced *Streptococcus thermophilus* strains. (TIFF)
