# Supplementary figures and images for: Streptococcus thermophilus Biofilm Formation: A Remnant Trait of Ancestral Commensal Life?
Source: PLoS One. 2015 Jun 2;10(6):e0128099. doi: 10.1371/journal.pone.0128099 (PMC4452758; doi:10.1371/journal.pone.0128099)

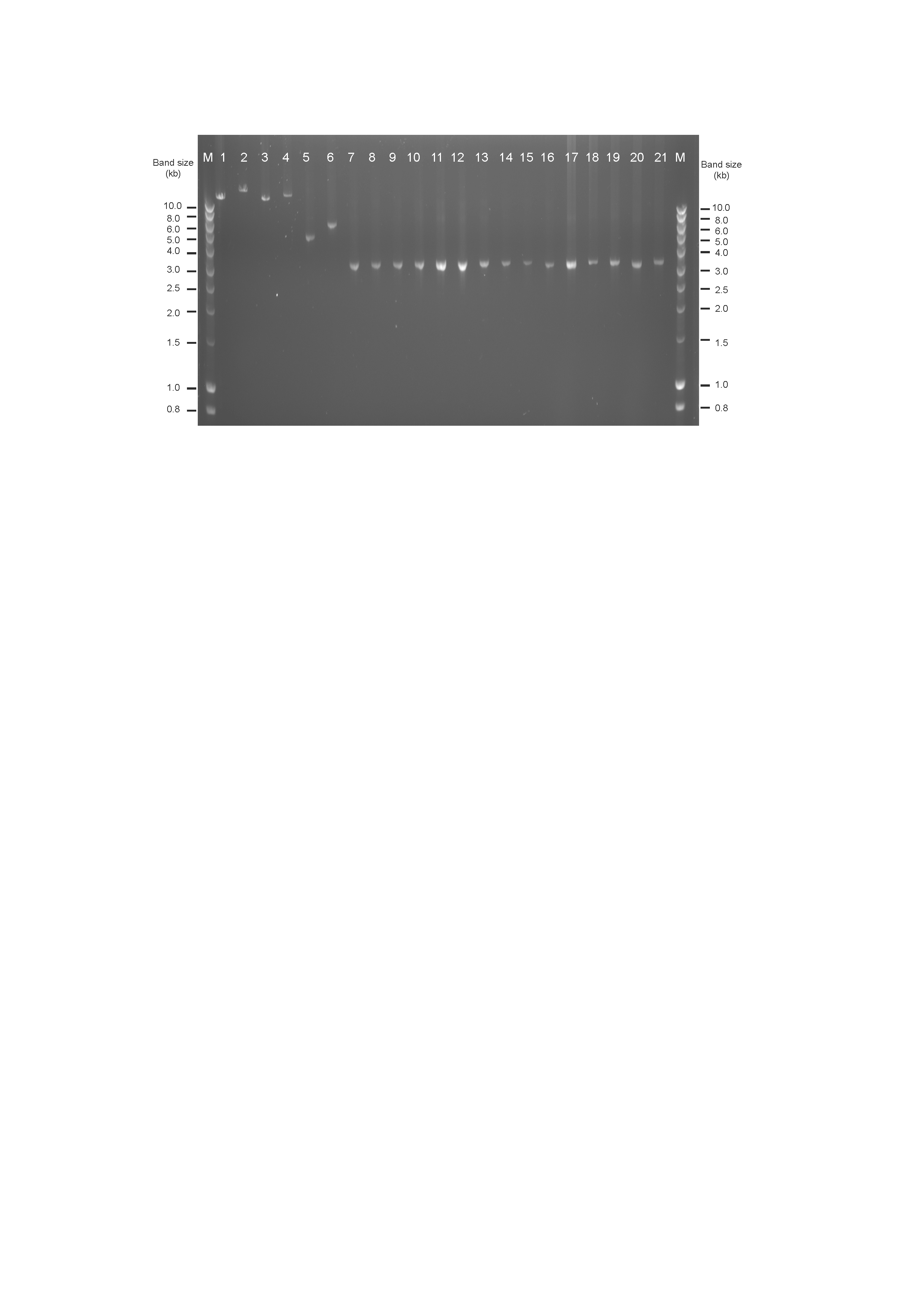

Supplement: S2 Fig — The genomic region comprised between metK (STH8232_1369) and murA1 (STH8232_1353) was amplified by PCR from genomic DNA of S. thermophilus strains with the MURA1 and METK primers. Numbers on the top of the electrophoresis gel lanes referred to the name strains as following: M, marker; 1, LMD-9; 2, JIM8232; 3, JIM10010; 4, JIM10116; 5, JIM10032; 6, JIM10119; 7, JIM10001; 8, JIM10020; 9, JIM10031; 10, JIM10037; 11, JIM10050; 12, JIM10055; 13, JIM10087; 14, JIM10100; 15, JIM10114; 16, JIM10117; 17, LMG18311; 18, CNRZ1066; 19, CNRZ759; 20, CNRZ1575; 21, CNRZ1595. (TIFF) [file pone.0128099.s002.tiff]

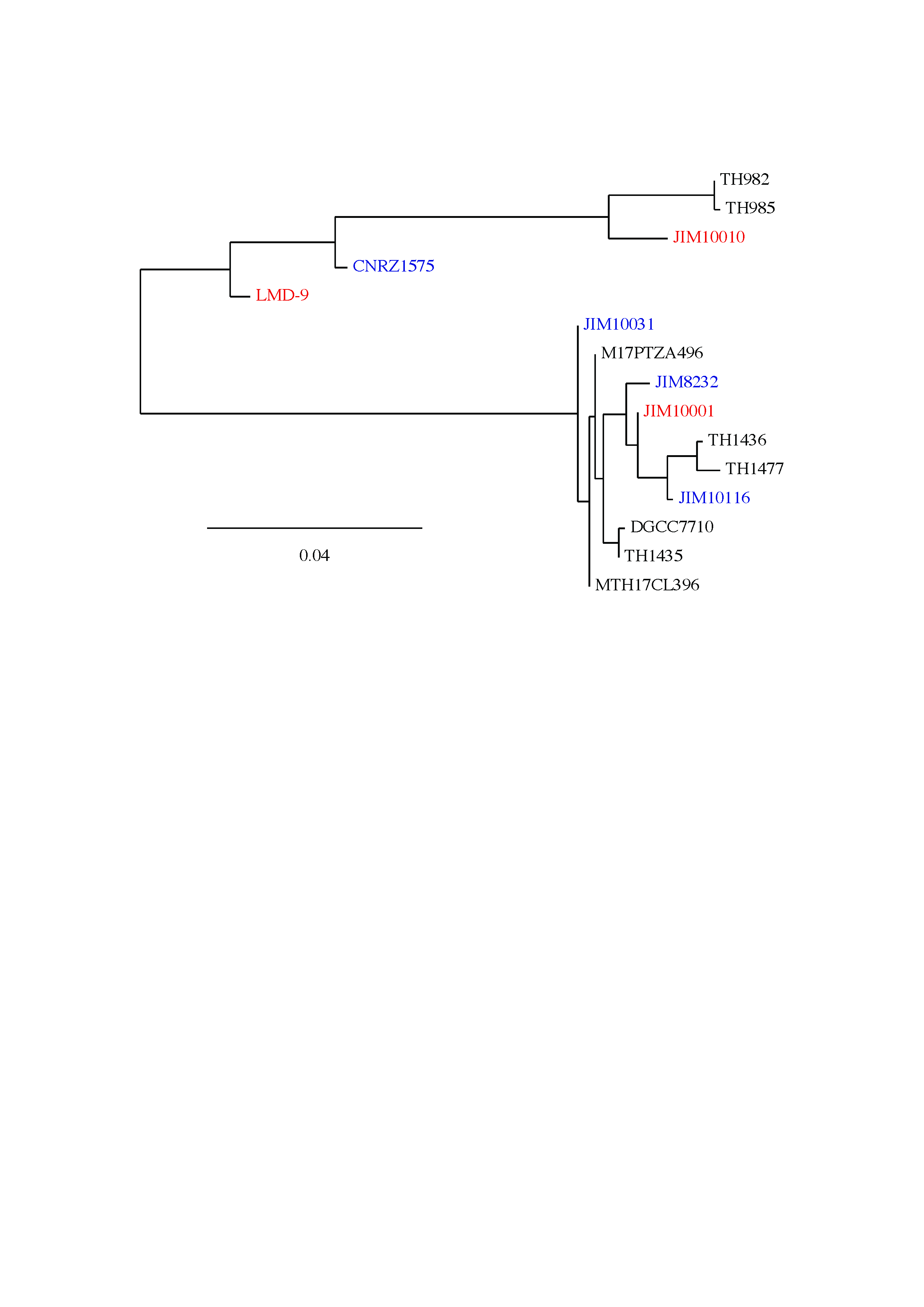

Supplement: S3 Fig — Blue, moderate and strong biofilm producing strains; Red, no or poor biofilm producing strains; Black, strains for which biofilm formation ability was not determined. (TIFF) [file pone.0128099.s003.tiff]

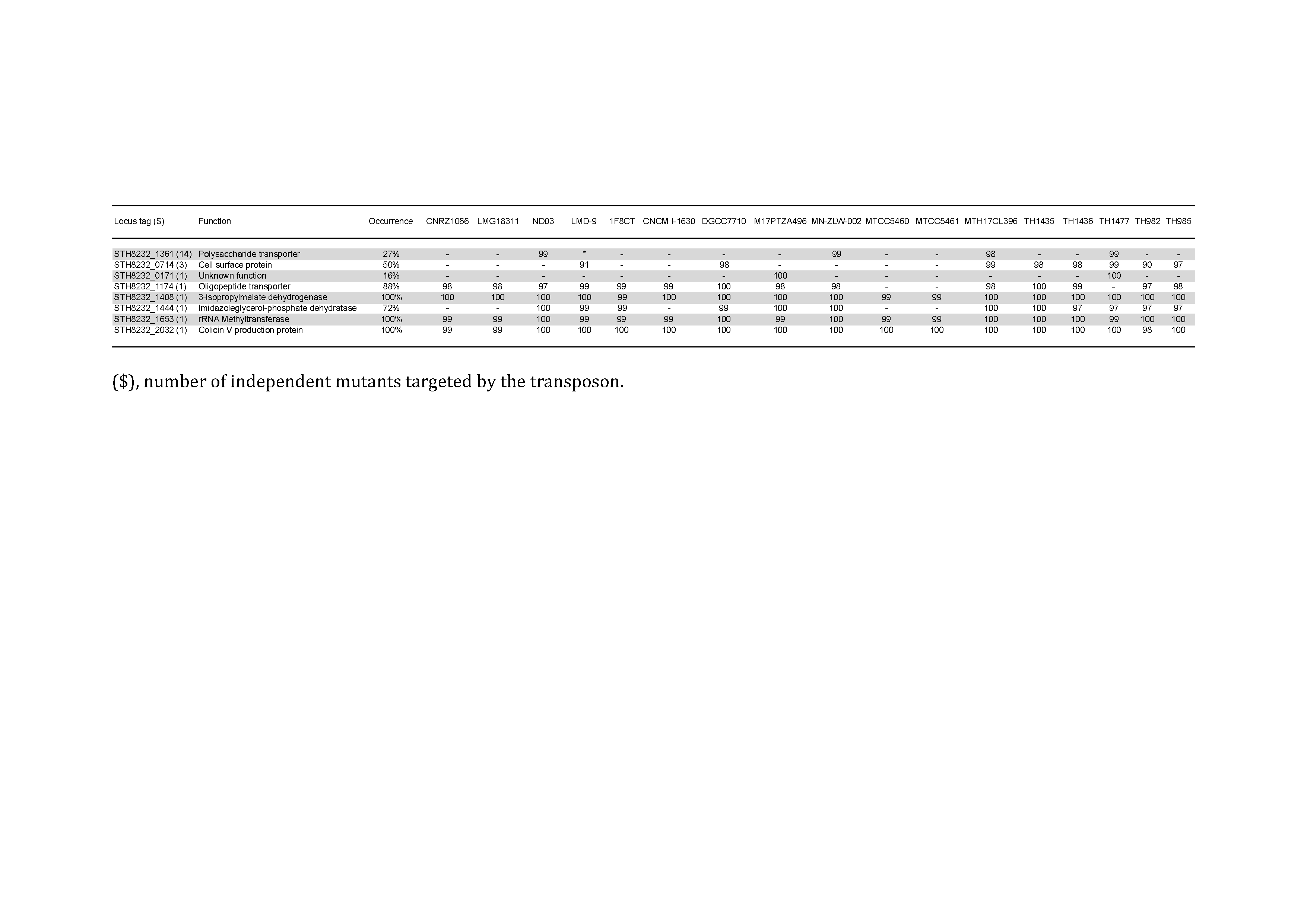

Supplement: S2 Table — (TIFF) [file pone.0128099.s005.tiff]
